# Predictors of Life Satisfaction in New Zealand: Analysis of a National Dataset

**DOI:** 10.3390/ijerph19095612

**Published:** 2022-05-05

**Authors:** Rebecca J. Jarden, Mohsen Joshanloo, Dan Weijers, Margaret H. Sandham, Aaron J. Jarden

**Affiliations:** 1 Department of Nursing, Melbourne School of Health Sciences, The University of Melbourne, 161 Barry St., Carlton, VIC 3053, Australia; 2 Department of Psychology, Keimyung University, 1095 Dalgubeol Boulevard, Dalseo-Gu, Daegu 42601, Korea; mjoshanloo@gmail.com; 3 School of Social Sciences, University of Waikato, Gate 1 Knighton Road, Private Bag 3105, Hamilton 3240, New Zealand; dan.weijers@waikato.ac.nz; 4 School of Clinical Sciences, Auckland University of Technology (AUT), North Shore Campus, 90 Akoranga Drive, Northcote, Auckland 0627, New Zealand; margaret.sandham@aut.ac.nz; 5 Centre for Wellbeing Science, Melbourne Graduate School of Education, The University of Melbourne, Kwong Lee Dow Building, 234 Queensberry Street, Parkville, Melbourne, VIC 3053, Australia; aaron.jarden@unimelb.edu.au

**Keywords:** Gallup World Poll, life satisfaction, New Zealand, wellbeing

## Abstract

The study aim was to determine prevalence and predictors of life satisfaction in New Zealand. In this observational cross-sectional study, a sample of 10,799 participants from NZ were drawn from the Gallup World Poll from 2006 to 2017. Data were analysed using regression analysis and ANOVA. Prevalence of life satisfaction across time varied little from a high of 7.61 (*SD* = 1.6) in 2007 to a low of 7.23 (*SD* = 1.73) in 2011 (range 0–10). Satisfaction with standards of living predicted life satisfaction regardless of age or gender. For males across all age groups and females up to age 40 years, positive experiences and satisfaction with household income were important predictors. Being married was an important predictor for males over 40 years and feeling satisfied with their current city was important for females across all ages and for men under 40. The levels of life satisfaction changed over time, possibly due to major national events. Satisfaction with standards of living was found to predict life satisfaction regardless of age or gender. These results provide a path for policy focus towards increased life satisfaction.

## 1. Introduction

### 1.1. Wellbeing Is Important to New Zealand

In 2018, the Prime Minister of New Zealand (NZ) announced that NZ would lead the world by embedding wellbeing into its budget decision-making process [1]. The core indicators covered social, cultural and environmental outcomes, aligning with the United Nations sustainable development goals [2]. Alongside the Prime Minister’s announcement, an updated version of NZ’s Living Standards Framework (LSF) was released [3]. Inspired by the Organisation for Economic Co-operation and Development’s “How’s Life?” approach [4], NZ’s new model of wellbeing for measuring national progress and guiding public policy included subjective wellbeing as a wellbeing output domain. In doing so, NZ signaled the importance of people’s subjective opinions about how their life is going.

### 1.2. Changes in New Zealand 2006 to 2017

Against the backdrop of increased policy focus on wellbeing, New Zealand faced notable social and economic events in the 2006–2017 time period, including two major earthquakes in the Canterbury region (September 2010 and February 2011) causing mass casualties and loss of homes and livelihoods; the Global Financial Crisis (GFC) causing a long recession [5,6] (approx. mid 2007 to early 2009); a large rise in unemployment levels from a record low of 3.7% in 2007 to 7% in 2009 [7,8]; and in 2008, New Zealand and China signed a historic free-trade agreement which led to a quadrupling of exports to China and an influx of Chinese investment, tourism, and students [9].

### 1.3. What Is Life Satisfaction?

One of the three measures of subjective wellbeing used in NZ’s Living Standards Framework is life satisfaction. Life satisfaction, or ‘satisfaction with life’, has been defined as a cognitive evaluation of overall satisfaction with an individual’s current life, relative to the individual’s own criteria of what a satisfactory life is [10]. Life satisfaction is widely regarded as a key measure of subjective wellbeing [4,11,12], and is one of the most predominant measures of wellbeing per se. Measures of life satisfaction are appealing to policy makers because they are understandable and have been, and are still, used extensively in international surveys [13]. Life satisfaction measures are subjective global assessments—they assess respondents’ own views of how their life is going for them. The measures are highly subjective in that respondents must draw on their own individual views of what is important in life to judge how their life measures up. This ability to incorporate a wide range of views on what is important in life is a key reason for some researchers to view life satisfaction as the most important measure of subjective wellbeing [14]. Individual life satisfaction items have demonstrated consistently high correlations with a broad range of much more complex measures of wellbeing, including objective measures [4,14,15,16,17,18].

### 1.4. Life Satisfaction in New Zealand

Few life satisfaction studies have been undertaken within New Zealand at national level. The Christchurch Health and Development longitudinal birth cohort study [19] evaluated life satisfaction in relation to mental health. A reciprocal association was found between mental health problems and life satisfaction, with the study concluding that life satisfaction influences mental disorder, and mental disorder influences life satisfaction. For university medical students, life satisfaction was found to be negatively correlated with anxiety and depression [20]. Māori and European New Zealanders demonstrate differences in the strength of the relationship between life satisfaction and work–life balance [21]. For Māori, higher levels of work–life balance were not associated with higher levels of life satisfaction, but for European New Zealanders, they were. These differences were attributed to Māori tending to be less individualistic than NZ Europeans, whose perceptions of overall quality of work and life experiences may be more strongly influenced by their perceptions of work–life balance [21,22]. Statistics New Zealand conducted a wellbeing survey of 5549 Māori aged over 15 years in 2013, which included Māori-specific measures of wellbeing in addition to life satisfaction and other variables [23]. Predictors of life satisfaction for Māori were reported as similar to international predictors. For example, demographic factors, such as age, sex, urban area, and marital status, predicted life satisfaction [23]. The subjective variables “adequacy of income; number of housing problems; health status; loneliness; trust in people; trust in courts; and importance of culture” were also significant predictors of life satisfaction [23].

Most of the existing national studies of the predictors of life satisfaction in NZ have used data from the General Social Survey. Using data from the 2008 wave of the General Social Survey, Brown, Woolf, and Smith [12] found that the main international trends were also present in NZ. In particular, they found the strongest predictors of life satisfaction to be income, unemployment, health status (especially mental health), and social contact. Jia and Smith [24] found similar results using data from the 2009, 2010, and 2012 waves of the General Social Survey. In particular, they found that, when controlling for demographic variables, mental health, unemployment, and having someone to rely on in a crisis were the strongest predictors of life satisfaction (p. 15). More than the previous study, this one emphasized that although “income is highly significant and positively related to life satisfaction…, the potential impact is small” (pp. 15–16). Although the international literature generally finds the income–life satisfaction relationship to be small, Jia and Smith [24] used the Sacks et al. [25] estimate of the relationship found internationally to show that income is probably a much weaker determinant of life satisfaction in NZ. Another NZ study, the Sovereign New Zealand Wellbeing Index [26], investigated 10,000+ New Zealanders’ wellbeing over time, identifying no real change in wellbeing between 2012 and 2014, and that a significant predictor of wellbeing was living comfortably on present income. The authors concluded that: “While earning more money isn’t always a realistic option, evaluating how you are living within your means is an important consideration for your wellbeing” (p. 15).

The aforementioned studies produced results in line with international expectations for the relationships between demographic variables and life satisfaction [24]. Females reported slightly higher averages of life satisfaction throughout the life course than males, with both groups reaching a nadir about 45 years old [24,27]. Being partnered or married (i.e., in a relationship) was also a significant predictor of life satisfaction in both studies. Measures of community trust, engagement, and safety were all correlated with life satisfaction in both studies, but the coefficients of most were small [12,24,28]. Education results were partially mixed between the studies, but generally weak or insignificant when other variables were controlled for [12,24,29]. Being unemployed tends to cause a significant drop in life satisfaction [30], even after controlling for the associated reduction in income [31,32]. Taken as a whole, the above studies suggest that NZ is not that different to its Western counterparts when it comes to life satisfaction, regarding both the prevalence and predictors of it.

However, some variables that have been found to predict life satisfaction have not been assessed in the studies of subjective wellbeing in NZ. Country of origin has been shown to predict life satisfaction, especially when the cultures of the current and original nations are very different [33,34]. Religious affiliation has been found to predict life satisfaction in some international studies [35,36] and in New Zealand [37]. However, the relationship appears complex, and can disappear when controls are added [37]. Given the complexity of the relationship and the (decreasing, but) sizable importance of religion in New Zealand [38], further investigation seems warranted.

### 1.5. Study Objectives

“What predicts life satisfaction, and to what extent?” is likely to be influenced by cultural differences and major events over time. Having detailed analyses of the prevalence and predictors of life satisfaction in as many nations as possible could explicate cultural understanding of differences in correlates of life satisfaction whilst also providing insight into the specific nation under study [39]. Since, as mentioned, there have only been a few national surveys of the predictors of life satisfaction in NZ, the objectives of the present study are to report a national investigation of the prevalence and predictors of life satisfaction in NZ and whether, and to what extent, the predictors commonly found in international research play a similar role in NZ. Special attention is paid to the most predictive variables and those that our data can shed more light on than the previously published studies: age, sex, income, relationship status, and employment status.

## 2. Materials and Methods

### 2.1. Study Sample

**Figure 1:**
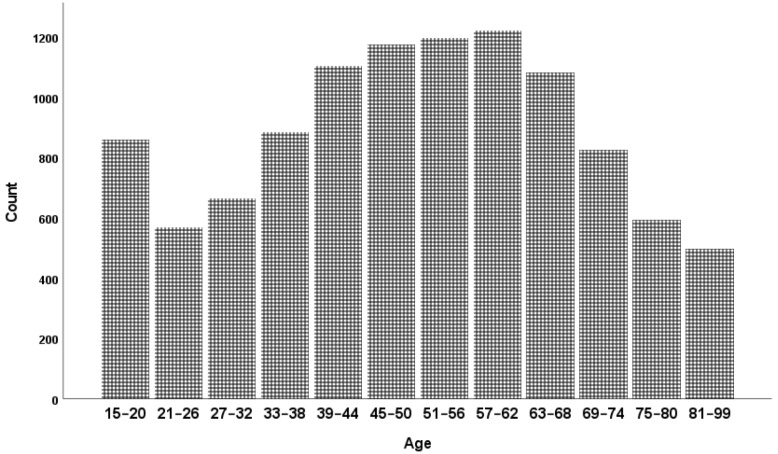
Age distribution (*N* = 10,799).

The data came from the Gallup World Poll, which has collected nationally representative samples from NZ since 2006. Each year, randomly selected participants (aged 15 and older) have been contacted via landline and/or mobile telephones for participation in the survey. New Zealand sample sizes are approximately 500 to 2000 for each year, with an average of 982 per year across international data. We used all available data from 2006 to 2017 in the present analyses, consisting of 10,799 participants (57.4% females, *M*_age_ = 50.174, *SD*_age_ = 18.655) which is an average of 900 across the waves. The age distribution of all participants across all years (2006 to 2017) is shown in Figure 1.

### 2.2. Measures

We used several items from the battery of Gallup World Poll items including the seven demographic variables of: employment, education, location, religious affiliation, relationship status, country of birth, and income quintile. These items measure variables that have been identified as relevant predictors of life satisfaction as discussed above. The items and their response formats are presented in Supplementary Materials (Table S1).

#### Life Satisfaction

The Ladder of Life Scale [40] was used to measure life satisfaction. This scale asks participants to:

“Please imagine a ladder with steps numbered from zero at the bottom to ten at the top. The top of the ladder represents the best possible life for you and the bottom of the ladder represents the worst possible life for you. On which step of the ladder would you say you personally feel you stand at this time?”

Reliability and validity for this scale have been reported as adequate [41].

### 2.3. Analysis

Intercorrelations between items were weak; so, all items were used separately as variables. However, using factor analysis, we were able to calculate two composite variables. The results of a principal axis factoring with six affective variables showed that a two-factor structure was consistent with the data, as only two of the eigenvalues were above 1.0 (i.e., 2.289 and 1.140). The factor analysis was repeated with a promax rotation to obtain rotated factor loadings. Only loadings greater than 0.4 were considered nontrivial. Stress, worry, sadness, and anger had nontrivial loadings (ranging from 0.416 to 0.788) on the first factor (variance explained = 28.330%). These four variables were averaged to form an index of negative experience (Kuder–Richardson 21 reliability coefficient = 0.589). Factor 2 had only two nontrivial loadings (both = 0.646), laughter and enjoyment (variance explained = 9.513%). The two items for positive affect were averaged to form an index of positive experience (Kuder–Richardson 21 reliability coefficient = 0.592). Data were analysed using regression analysis to examine which variables significantly predict life satisfaction and ANOVA to examine group differences in life satisfaction. We used a standard or simultaneous regression model. In this model, all predictors enter the regression equation simultaneously; each is evaluated as if it entered the regression after all other predictors. In other words, each predictor is evaluated based on its unique contribution to predicting dependent variables, after other predictors’ contributions are controlled for [42]. We used stepwise regression as a supplementary tool to filter out the best predictors from our long list of potential predictors. Stepwise regression helps reduce a long list of potential predictors to a manageable number of significant predictors to facilitate interpretation. While the simultaneous method retains all entered variables regardless of significance level, this method combines the forward and backward approaches to remove nonsignificant predictors. The variance explained by each predictor changes as more predictors are added to the equation. As more predictors enter the equation, a variable that has qualified for inclusion may lose some of its predictive power. In this case, the variable with “weakened” predictive power is removed using the stepwise procedure [43].

### 2.4. Ethical Considerations

This study used publicly available data and as such did not require further ethical approvals.

## 3. Results

### 3.1. Prevalence of Life Satisfaction over Time

**Figure 2:**
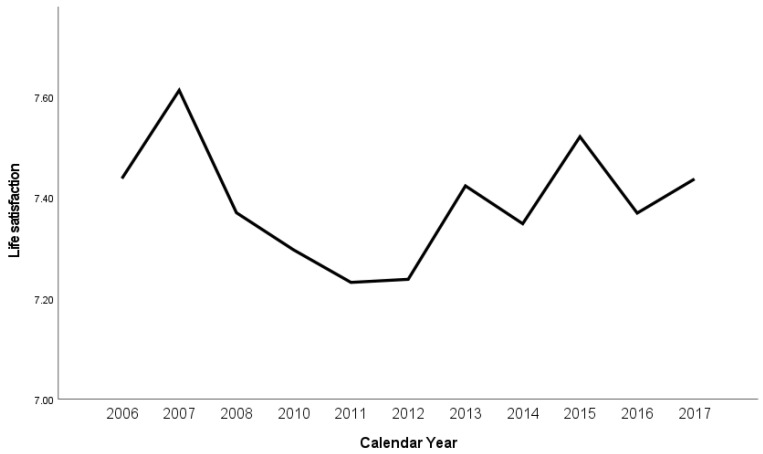
Prevalence of life satisfaction in New Zealand from 2006 to 2017.

Life satisfaction over time (years 2006 to 2017) is illustrated in Figure 2 and full descriptive statistics can be found in Supplementary Materials (Table S2).

Between 2006–2017 mean life satisfaction varied from a high of 7.61 (*SD* = 1.6) in 2007 to a low of 7.23 (*SD* = 1.73) in 2011; the range in mean changing 0.38 over this time between the high and low.

### 3.2. Life Satisfaction by Age and Gender

**Figure 3:**
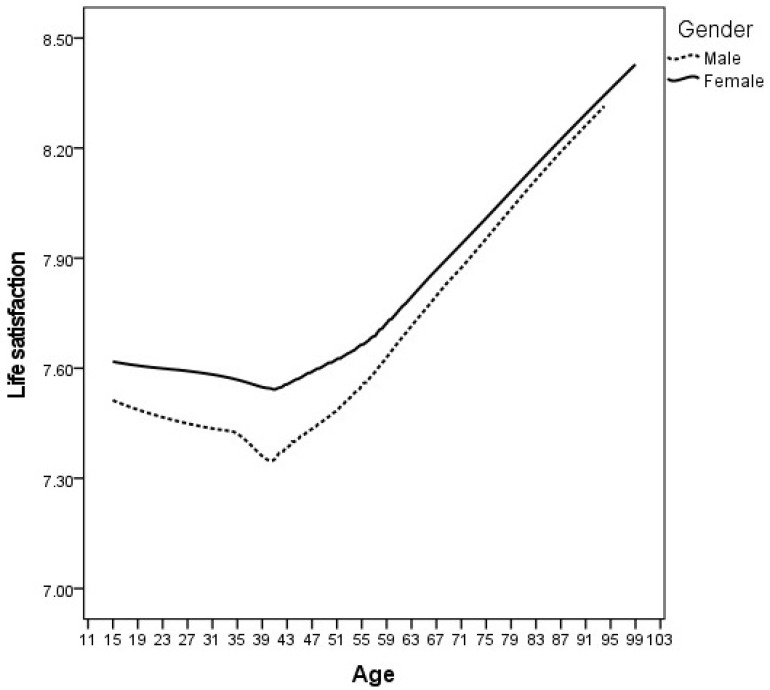
Life satisfaction by age and gender.

The results of an independent samples *t*-test showed that women scored significantly higher than men on life satisfaction (*t*(10,771) = −3.763, *p* < 0.001, 95% CI of difference: −0.186, −0.058, *d* = 0.073), albeit with a very small effect size. Figure 3 shows the distribution of life satisfaction by age and gender for all participants across all years.

Figure 3 shows that females tend to report being slightly more satisfied with life throughout and over the life course compared to males, with this gender gap being most predominant mid-life. Both females and males on average report slightly decreasing levels of life satisfaction from teenage to mid-life, and then increasing from mid-life onwards.

As a supplementary analysis, we also looked at gender differences in the positive and negative experience indexes we created (composite variables described above). The results of independent samples *t*-tests showed that women scored significantly higher on the negative experience index (*t*(9401.196) = −7.238, *p* < 0.001, 95% CI of difference: −0.054, −0.031, *d* = 0.146), again with a small effect size. No significant gender differences were found for the positive experience index.

### 3.3. Other Demographic Predictors of Life Satisfaction

Table 1 presents the results of seven separate ANOVAs, using demographic variables as independent variables (employment, education, location, religious affiliation, relationship status, country of birth, income quintile) explaining life satisfaction.

**Figure 4:**
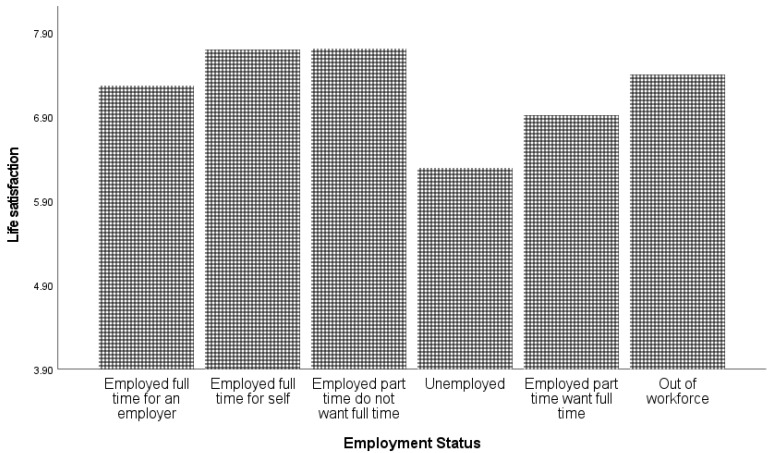
Life satisfaction and employment status.

For religious affiliation, several categories with very small sample sizes (e.g., “Hinduism” and “Islam”) were combined with the “other” category. As indicated in Table 1, the strongest predictor of life satisfaction was income quintile (explaining 3.7% of the variance), followed by employment status (explaining 2.9% of the variance) and relationship status (explaining 2.5% of the variance). Figure 4 presents life satisfaction and employment status.

The self-employed and those who choose to work part-time had the highest levels of life satisfaction and those unemployed experienced the lowest levels of life satisfaction. The results of the Games–Howell test showed that all employment groups were significantly different from each other (*p* < 0.05) except employed full-time for an employer and out of workforce (*p* = 0.074).

**Figure 5:**
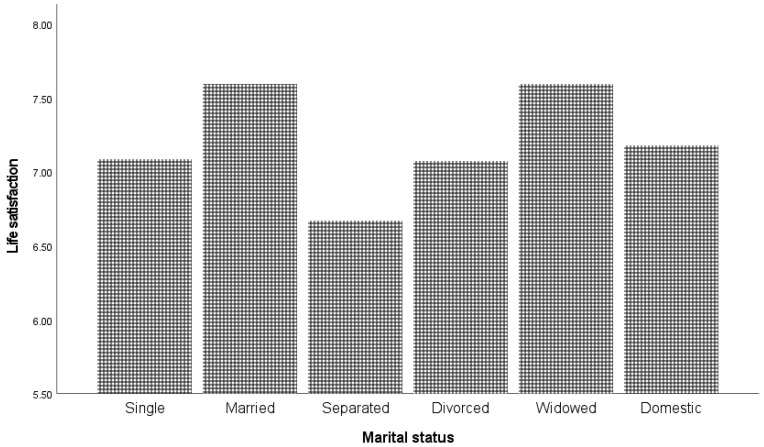
Life satisfaction and relationship status.

Figure 5 presents life satisfaction and relationship status.

Individuals identifying as married or widowed reported the highest levels of life satisfaction, and those identifying as separated reported the lowest levels of life satisfaction. In summary, and as shown in Figure 4 and Figure 5 and Table 1, the unemployed and separated groups of individuals were in comparison the least satisfied with their lives. The results of the Games–Howell test showed that all marital groups were significantly different from each other (*p* < 0.05) except single and domestic partnership (*p* = 0.717) and domestic partnership and divorced (*p* = 0.856). A separate ANOVA indicated that gender did not moderate the relationship between relationship status and life satisfaction. The results of the Games–Howell test showed that all income groups were significantly different from each other (*p* < 0.05). Education, religious affiliation, and location each explained 0.7% or less of the variance in life satisfaction. Country of birth was not a significant predictor. The results of the Games–Howell test showed that there was a significant difference between people with elementary and tertiary education, and between people with secondary and tertiary education (*p* < 0.01). For religious affiliation, the Games–Howell test showed that all groups were significantly different from each other (*p* < 0.01), whereas for location, the significant differences were between “rural or farm” and all other types of location, as well as between “small town or village” and “Suburb of a large city” (*p* < 0.01).

### 3.4. Regression Analysis

We included all predictors of life satisfaction along with key demographic variables (the variables are provided in the predictor column of Table 2) in a regression using the standard or simultaneous regression model. A total sample of 6023 participants, of the 10,799 participants (56%), had no missing values on all of the 28 variables and were included in the analysis (see Table 2).

The predictors collectively explained 33.1% of the variance in life satisfaction, *F*(27, 5995) = 110.024, *p* < 0.001, *R*^2^ = 0.331. Based on the results of a separate stepwise regression analysis, household income satisfaction was the strongest predictor explaining 16.8% of the variance. The second strongest predictor was satisfaction with standards of living, contributing an additional 5.4%. Negative experience, city satisfaction, and positive experience were next explaining 3.4%, 1.9%, and 1.7%, respectively. These five variables jointly explained 29.3% of the variance in life satisfaction scores. The other variables collectively added only 3.8% of additional variance. Based on the results of the stepwise regression, perceptions of corruption, donation, religiosity, respect, education, separated, unemployed, and number of children did not add a significant amount of variance beyond the other 19 variables and were excluded.

We also conducted regression analyses separately across age and gender groups. Given the large sample size, the probability of a type 1 error is increased. Therefore, the significance threshold of 0.001 is preferred for assessing significance. Table 3 presents the unstandardised regression coefficients for age and gender groups.

Satisfaction with healthcare, being separated, and unemployed were removed as they showed no variation in one or more groups. Table 4 presents the regression results across age and gender groups, and also presents the five most important predictors for each group in order of predictive power, based on separate regression analyses using the stepwise procedure for each group.

For all groups, an important predictor of life satisfaction was satisfaction with standards of living. For males across all age groups and females up to the age of 40 years, positive experiences and satisfaction with household income were also important predictors. Being married was an important predictor for males over 40 years. Feeling satisfied with city was important for females across all ages and for men under 40.

### 3.5. Relationship between Household Income and Life Satisfaction: A Close Examination

**Figure 6:**
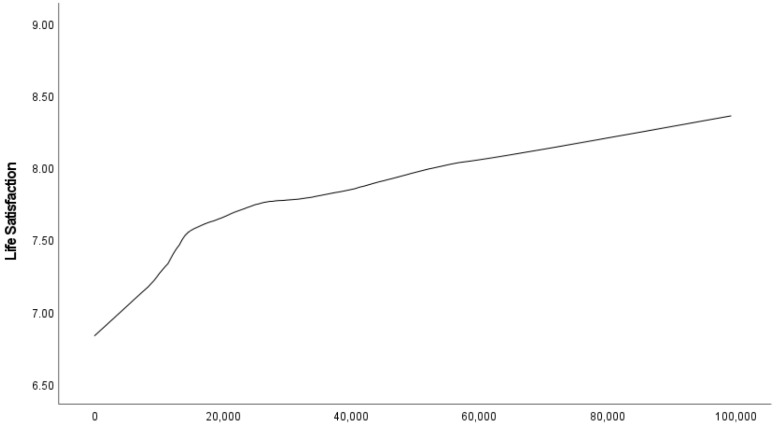
The relationship between annual household income (in International Dollars) and life satisfaction in NZ.

The relationship between per capita annual household income in International Dollars, e.g., see [44], and life satisfaction is shown in Figure 6.

Individuals with incomes over $100,000 International Dollars were excluded due to their small sample size. As can be seen, the lower income group (under $15,000 International Dollars, equivalent to about $23,000 NZD) reported much lower life satisfaction, however, past approximately $20,000 International Dollars (equivalent to about $31,000 NZD), there were smaller increases as income increased.

## 4. Discussion

### 4.1. Prevalence of Life Satisfaction

Life satisfaction research has typically comprised cross-sectional observational studies correlating various demographic, economic, health, education, social and community, and personality factors with life satisfaction. In this study, we have extended this research to both prevalence and predictors for NZ people. Regarding the prevalence of life satisfaction in the NZ population between 2006–2017, we found small changes over time from a high of 7.61 (*SD* = 1.6) in 2007 to a low of 7.23 (*SD* = 1.73) in 2011 [7]. This result indicates that NZ is consistently higher than the world means reported in the World Happiness Reports across time, for example, from 2006 to 2015, these range from 4.45/10 to 5.3/10 (e.g., see [16,45,46]). Notably, the NZ low of 7.23 reported for 2011 occurs around the time of the significant earthquakes in the major NZ city of Christchurch at the end of 2010 and beginning of 2011. These earthquakes had devastating and ongoing impacts locally and nationally, which not only created immediate illbeing and traumatic impacts in the Christchurch region, but also created stress and anxiety nationally [47]. Also notable in this time period was the Global Financial Crisis (GFC) of 2007–2008 which negatively impacted many New Zealanders through the shrinking of the Gross Domestic Product (GDP) over five consecutive quarters [5]. Unemployment in 2007 was at a record low, but the GFC that followed contributed to the rise in unemployment back to 7% in late 2009 [7]. In sum, while the prevalence of life satisfaction in NZ was higher than the global average [45], there was a small amount of change during the study period in reported life satisfaction, which may be attributed to some extent to the major events that happened in NZ during that time period.

### 4.2. Predictors of Life Satisfaction

The existing literature holds mixed results for the relationship between gender and life satisfaction. Various studies reported a range of relationships, including men being more satisfied by a small amount, men and women being about as satisfied, and, as we have here, women being slightly more satisfied with their lives (see [48]). Donovan and Halpern [13] suggested that men may be under-reporting their emotional experiences. Regardless, it is becoming increasingly accepted that women tend to report higher levels of life satisfaction when compared to men of the same age [49]. Although the reasons have not yet been thoroughly explored, the modest “U-shaped” relationship between age and life satisfaction in our results matches what has been found in many studies [13,50].

We found for all groups, across both age and gender, satisfaction with standards of living was the most important predictor of life satisfaction, and satisfaction with household income was the second or third most important predictor. There is most likely a direct relationship between these two variables, as satisfaction with household income is likely to impact satisfaction with standards of living. As can be seen in Figure 6, (objective) household income also seems to impact life satisfaction, especially for those with very low incomes. Studies using similar methodologies demonstrate the same income-related findings in a diverse range of nations, including Italy [51] and the United Arab Emirates [52]. The relationship between life satisfaction and income has been well studied, but is complex [53,54]. People from wealthier countries are more satisfied than citizens of poorer countries, and within a country, richer people are more satisfied with life than poorer people [55]. Yet, it appears that relative income matters for life satisfaction, and that habituation occurs as people adjust to levelling up through income brackets [12,56].

We found being married significantly predicted life satisfaction when looking at the whole population. This is in line with existing results for Australasia (and Western nations generally), which usually show that being married is positively associated with life satisfaction, and being divorced, widowed or separated is negatively associated with wellbeing [57]. However, age group analysis revealed that the association between being married and life satisfaction was driven by the older age group. Being married was a significant predictor for females over 40 years old and a significant and important predictor for males in the same age group. However, being married was an insignificant predictor for males and females under 40 years of age.

Education has been associated with life satisfaction, but the majority of the variance can be explained by differences in income, health, and social capital [49]. Life satisfaction is greater amongst people with high physical and mental health, and subjective evaluations of health status are more strongly correlated with subjective wellbeing than objective measures [58]. Our results are in line with these findings. Education was not a significant predictor of life satisfaction, but subjective measures of income satisfaction, health problems, and negative emotions all were, with negative emotions being an important predictor and income satisfaction being the most important. Our more detailed analysis shows that income satisfaction is an important predictor for both age groups for males and females, and that negative experiences are also an important predictor for all groups except females under 40 years of age.

The importance of positive affect for life satisfaction has been stressed by Frederickson [59]. Our results reflect this. Positive experience, which contains items related to positive affect, was a significant predictor of life satisfaction. As shown in Table 4, positive experience was an important predictor of life satisfaction for males and females under 40 years old and males over 40 years old. It is unclear why positive experience was not an important predictor of life satisfaction for females over 40 years old in our study. Despite sex differences in life satisfaction found here and elsewhere, and the obvious differences in experiences and expectations across age groups, very little research has investigated the importance of positive and negative affect for life satisfaction across these different groups [60]. Future research should investigate this aspect in more detail.

In our study, feeling satisfied with city was important for females in both age groups and for men under 40. Perhaps people in these groups find it important to have suitable options for activities outside of their home. Life satisfaction has also been related to environmental factors in NZ. For example, residents with easy access to greenspace in their neighborhood reported higher levels of life satisfaction; however, this effect is nearly eliminated when the person has a high fear of crime in their neighborhood [61]. Feeling safe at night was not predictive of life satisfaction in our study. However, the evidence on the relationship between life satisfaction and with security and safety is somewhat patchy and mixed [49], but several recent studies do find it to be significant, albeit small [62,63,64].

The importance of satisfaction with living standards and household income, and the strong relationship between income and life satisfaction for the lowest income New Zealanders, suggests that improvements to the average life satisfaction of New Zealanders might be achieved by securing high incomes and standards of living for the worst off in society. While such policies generally require greater government intervention, international studies suggest that nations with greater income redistribution and social services have higher life satisfaction [65,66]. The minimum wage in NZ has increased recently, but further policies that increase incomes or living standards for the poorest New Zealanders may be required to address societal issues such as child poverty [67,68]. Alternatively, as the Sovereign study suggested [26], learning to live within your means, regardless of income level, seems to positively impact wellbeing. For example, in the Sovereign study, the odds ratio of having very high wellbeing was 12 times higher for people living within their means compared to people finding it difficult to live with their present income.

### 4.3. Strengths and Limitations

Jebb and colleagues noted limitations in studies assessing life satisfaction, including that life satisfaction is a cognitive assessment of happiness, which overlooks that the subjective wellbeing construct also contains positive and negative affect [69]. Instead of including affect in our outcome measure, we assessed positive and negative experiences in the present study (through affective items about emotions and enjoyment). Positive and negative experiences were found to be important predictors of life satisfaction; so, they were still an important part of the study.

Another potential limitation is that, despite analysing a wide range of variables, the variables could not (even collectively) explain the majority of variance in life satisfaction. Furthermore, small effect sizes were found suggesting that whilst a statistical relationship was significant, the real-world impact of these findings may be small. This is to be expected. Previous research demonstrates the important contributions of genetics, behaviour, and personality to life satisfaction (e.g., see [70,71]). In the current study, we used demographic and other variables that are more closely related to the levers of policy in order to provide insights that are potentially useful for the generation of wellbeing policy recommendations. Further exploration of the relationships between income, satisfaction with income, and life satisfaction, as well as an individual’s relationships to and perceptions of income remain a future research opportunity.

## 5. Conclusions

Life satisfaction in NZ changed slightly over the study period, most likely due to major national and international events such as the Christchurch earthquakes and the GFC. Satisfaction with standards of living was found to be an important predictor of life satisfaction regardless of age or gender. These findings may provide a path for policy focus directed towards raising standards of living which will in turn increase life satisfaction. Policy may also be used to respond to the increasing social discourse on inequalities between income levels, genders and age groups which has been growing for some time in NZ and impacts life satisfaction. In line with other policy recommendations based on similar analyses internationally [28,72], Figure 6 implies that raising the living standards of New Zealanders with the lowest incomes might improve average life satisfaction more than raising them for those with the highest incomes.

## Figures and Tables

**Table 1:** ANOVA Results Predicting Life Satisfaction.

Independent Variable	Category	Raw Mean	*SD*	*N*
Employment	Employed full-time for an employer	7.273	1.516	3109
*df =* 5, 8251 *F* = 48.884 *p* < 0.001 *η*^2^ = 0.029	Employed full-time for self	7.696	1.564	706
	Employed part-time do not want full-time	7.710	1.434	1172
	Unemployed	6.294	2.004	285
	Employed part-time want full-time	6.917	1.806	484
	Out of workforce	7.397	1.853	2501
	Total	7.354	1.678	8257
Education	Elementary	7.276	1.986	1030
*df =* 2, 10,575 *F* = 12.231 *p* < 0.001 *η*^2^ = 0.002	Secondary	7.342	1.676	6563
	Tertiary (four years beyond high school)	7.506	1.525	2985
	Total	7.382	1.670	10,578
Location	Rural or farm	7.661	1.694	1827
*df =* 3, 10,718 *F* = 25.094 *p* < 0.001 *η*^2^ = 0.007	Small town or village	7.424	1.707	2469
	Large city	7.326	1.659	1308
	Suburb of a large city	7.275	1.638	5118
	Total	7.381	1.672	10,722
Religious affiliation	Christian	7.427	1.649	5198
*df =* 2, 8998 *F* = 17.820 *p* < 0.001 *η*^2^ = 0.004	Secular/Non-religious	7.301	1.690	3153
	Other	7.043	1.790	650
	Total	7.355	1.677	9001
Relationship status	Single	7.084	1.768	2557
*df =* 5, 10,717 *F* = 54.524 *p* < 0.001 *η*^2^ = 0.025	Married	7.589	1.542	5451
	Separated	6.670	1.867	282
	Divorced	7.074	1.804	674
	Widow	7.594	1.799	961
	Domestic partnership	7.177	1.589	798
	Total	7.382	1.672	10,723
Country of birth	Born in NZ	7.378	1.678	6618
*df =* 1, 8745 *F* = 0.244 *p* = 0.622 *η*^2^ = 0.000	Born in another country	7.357	1.662	2129
	Total	7.373	1.674	8747
Income quintile	Poorest 20%	6.818	1.963	1206
*df =* 4, 8252 *F* = 78.291 *p* < 0.001 *η*^2^ = 0.037	Second 20%	7.097	1.871	1542
	Middle 20%	7.313	1.611	1644
	Fourth 20%	7.483	1.508	1736
	Richest 20%	7.772	1.400	2129
	Total	7.354	1.679	8257

**Table 2:** Comprehensive Regression Analysis.

Predictor	*B*	95.0% CI for *B*		*t*	*p*	Beta
		Low	Up			
(Constant)	2.963	2.631	3.296	17.468	0.000	-
Female	0.262	0.186	0.338	6.754	0.000	0.077
Age	0.005	0.002	0.007	4.064	0.000	0.052
Squared age	0.000	0.000	0.000	4.911	0.000	0.059
Negative experience	−0.703	−0.839	−0.566	−10.084	0.000	−0.122
Positive experience	0.500	0.380	0.620	8.153	0.000	0.097
Health problems	−0.302	−0.394	−0.210	−6.424	0.000	−0.072
HH income satisfaction	0.405	0.351	0.458	14.755	0.000	0.191
Satisfaction with standards of living	0.762	0.645	0.880	12.720	0.000	0.162
Satisfied with healthcare	0.130	0.033	0.226	2.633	0.008	0.030
Satisfied with housing	0.115	0.043	0.187	3.139	0.002	0.034
Confidence in government	0.119	0.041	0.198	2.976	0.003	0.035
Corruption	−0.022	−0.130	0.087	−0.394	0.694	−0.005
City satisfaction	0.644	0.521	0.768	10.225	0.000	0.115
Helped	0.112	0.036	0.187	2.896	0.004	0.032
Volunteered	0.185	0.112	0.259	4.968	0.000	0.055
Donated	0.075	−0.008	0.157	1.778	0.076	0.020
Religiosity	−0.027	−0.104	0.050	−0.693	0.489	−0.008
Social support	0.435	0.271	0.599	5.199	0.000	0.057
Learned	0.175	0.095	0.256	4.292	0.000	0.049
Freedom	0.180	0.028	0.332	2.324	0.020	0.027
Safe at night	0.092	0.012	0.171	2.269	0.023	0.026
Respect	0.122	−0.023	0.268	1.648	0.099	0.019
Education	−0.026	−0.086	0.034	−0.848	0.396	−0.010
Separated	−0.139	−0.369	0.090	−1.194	0.233	−0.013
Married	0.255	0.176	0.335	6.313	0.000	0.076
Unemployed	−0.178	−0.372	0.015	−1.808	0.071	−0.020
No. of children	0.014	−0.026	0.054	0.689	0.491	0.008

Note. *B* = unstandardised regression coefficient. Beta = standardised regression coefficient.

**Table 3:** Unstandardised Regression Coefficients for Age and Gender Groups.

	Male		Female	
	15–39	40–99	15–39	40–99
(Constant)	3.667 ***	3.332 ***	3.506 ***	3.163 ***
Negative experience	−0.610	−0.816 ***	−0.428	−0.888 ***
Positive experience	0.643 ***	0.723 ***	0.462	0.348 ***
Health problems	0.021	−0.366 ***	−0.246	−0.200
HH income satisfaction	0.295 ***	0.465 ***	0.421 ***	0.432 ***
Satisfaction with standards of living	0.558	0.969 ***	0.538 ***	0.776 ***
Satisfied with housing	0.206	0.093	0.034	0.177
Confidence in government	0.175	0.085	0.018	0.105
Corruption	−0.040	−0.256	0.111	−0.010
City satisfaction	0.401	0.507 ***	0.538 ***	0.877 ***
Helped	0.126	0.097	0.085	0.007
Volunteered	0.064	0.123	0.282	0.255 ***
Donated	0.197	0.106	0.036	0.158
Religiosity	−0.087	0.024	−0.050	0.051
Social support	0.613	0.372	0.453	0.464 ***
Learned	0.102	0.184	0.207	0.212
Freedom	0.170	−0.229	0.348	0.408 ***
Safe at night	−0.049	0.010	0.129	0.057
Respect	0.233	0.105	0.502	−0.044
Education	−0.058	−0.021	−0.181	−0.027
Married	0.073	0.312 ***	0.190	0.180
Number of children	0.075	−0.042	−0.019	−0.023

Note. HH income = satisfaction with household income; *** *p* < 0.001. Given the large sample size, the probability of a type 1 error is increased. Therefore, the significance threshold of 0.001 is preferred for assessing significance.

**Table 4:** Regression Results across Age and Gender Groups.

		*R* ^2^	*F*	*df*	Most Important Predictors
Male	15–39	0.256	12.602 ***	21, 770	SWSL, Positive, HH income, Negative, City satisfaction
	40–99	0.350	51.077 ***	21, 1993	SWSL, Positive, HH income, Negative, Married
Female	15–39	0.274	19.333 ***	21, 1074	SWSL, Positive, HH income, City satisfaction, Respect
	40–99	0.341	69.680 ***	21, 2828	SWSL, HH income, Negative, City satisfaction, Volunteered

Note. The *R*^2^, *F*, and *df* values come from simultaneous regression analyses. The important predictors come from separate regression analyses using the stepwise method. The most important predictors are in order of predictive power. Abbreviations. SWSL = satisfaction with standards of living; HH income = satisfaction with household income; positive = positive experience; negative = negative experience. *** *p* < 0.001.

## Data Availability

Not applicable.

## Supplementary Materials

The following are available online at https://www.mdpi.com/article/10.3390/ijerph19095612/s1, Table S1: Items used in study, Table S2: Prevalence of life satisfaction in NZ from 2006 to 2017.
