# Supplementary material for: Predictors of Life Satisfaction in New Zealand: Analysis of a National Dataset
Source: Int J Environ Res Public Health. 2022 May 5;19(9):5612. doi: 10.3390/ijerph19095612 (PMC9103190; doi:10.3390/ijerph19095612)
Supplement: Supplementary file 1 [file ijerph-19-05612-s001.zip › ijerph-1647187-supplementary.pdf]

SUPPLEMENTARY FILES

Table S1. Items used in study

**Variable**	**Item**	**Response format**
Life satisfaction	Please imagine a ladder with steps numbered from zero at the bottom to ten at the top. The top of the ladder represents the best possible life for you and the bottom of the ladder represents the worst possible life for you. On which step of the ladder would you say you personally feel you stand at this time?	0 Worst possible to 10 Best possible
Enjoyment	Did you experience the following feelings during a lot of the day yesterday? How about Enjoyment?	1 Yes<br>2 No
Worry	Did you experience the following feelings during a lot of the day yesterday? How about Worry?	1 Yes<br>2 No
Sadness	Did you experience the following feelings during a lot of the day yesterday? How about Sadness?	1 Yes<br>2 No
Stress	Did you experience the following feelings during a lot of the day yesterday? How about Stress?	1 Yes<br>2 No
Anger	Did you experience the following feelings during a lot of the day yesterday? How about Anger?	1 Yes<br>2 No
Laughter	Did you smile or laugh a lot yesterday?	1 Yes<br>2 No
Freedom	In (this country), are you satisfied or dissatisfied with your freedom to choose what you do with your life?	1 Satisfied<br>2 Dissatisfied

Safe at night	Do you feel safe walking alone at night in the city or area where you live?	1 Yes<br>2 No
Respect	Were you treated with respect all day yesterday?	1 Yes<br>2 No
Learned	Did you learn or do something interesting yesterday?	1 Yes<br>2 No
Satisfaction with city	Are you satisfied or dissatisfied with the city or area where you live?	1 Satisfied<br>2 Dissatisfied
Household income satisfaction	Which one of these phrases comes closest to your own feelings about your household's income these days?	1 Living comfortably on present income<br>2 Getting by on present income<br>3 Finding it difficult on present income<br>4 Finding it very difficult on present income
Health problems	Do you have any health problems that prevent you from doing any of the things people your age normally can do?	1 Yes<br>2 No
Social support	If you were in trouble, do you have relatives or friends you can count on to help you whenever you need them, or not?	1 Yes<br>2 No
Satisfaction with standards of living	Are you satisfied or dissatisfied with your standard of living, all the things you can buy and do?	1 Satisfied<br>2 Dissatisfied

Donated	Have you done any of the following in the past month? Donated money to a charity.	1 Yes<br>2 No
Volunteered	Have you done any of the following in the past month? Volunteered your time to an organization	1 Yes<br>2 No
Helped	Have you done any of the following in the past month? Helped a stranger or someone you didn’t know who needed help	1 Yes<br>2 No
Corruption in Business	Is corruption widespread within businesses located in Korea, or not?	1 Yes<br>2 No
Corruption in Government	Is corruption widespread throughout the government in Korea, or not?	1 Yes<br>2 No
Born in NZ	Were you born in this country, or not?	1 Born in this country<br>2 Born in another country
Satisfaction with healthcare	In the city or area where you live, are you satisfied or dissatisfied with the availability of quality health care	1 Satisfied<br>2 Dissatisfied
Satisfaction with housing	In the city or area where you live, are you satisfied or dissatisfied with the availability of good affordable housing	1 Satisfied<br>2 Dissatisfied
Confidence in government	In (this country), do you have confidence in each of the following, or not? How about national government.	1 Yes<br>2 No
Religiosity	Is religion an important part of your daily life?	1 Yes<br>2 No

Number of children	How many children under 15 years of age are now living in your household?	
Note. All items had also two other response options: *Don’t know* and *Refuse to answer.* For the outcome variable and demographic variables, “Don’t know” and “Refused” were coded as missing. All other variables were dummy coded as 1 for “yes” or “satisfied” and 0 for “No”, “Dissatisfied”, “Don’t know” and “Refused”.		

Table S2. Prevalence of life satisfaction in NZ from 2006 to 2017

Year	*M*	*SD*	*N*
2006	7.438	1.677	1028
2007	7.612	1.595	750
2008	7.370	1.666	750
2010	7.296	1.721	750
2011	7.231	1.733	1000
2012	7.238	1.755	1008
2013	7.423	1.603	500
2014	7.348	1.683	2001
2015	7.520	1.557	1007
2016	7.369	1.674	1004
2017	7.436	1.647	1001
